# Parents’ Perceived Impact of the Societal Lockdown of COVID-19 on Family Well-Being and on the Emotional and Behavioral State of Walloon Belgian Children Aged 4 to 13 Years: An Exploratory Study

**DOI:** 10.5334/pb.1059

**Published:** 2021-06-29

**Authors:** C. Stassart, A. Wagener, A.-M. Etienne

**Affiliations:** 1 Inter University Health and Society Research Unit (URiSS), Health Psychology, Department of Psychology, University of Liege, Liege, Belgium

**Keywords:** COVID-19, societal lockdown, psychological impact, children

## Abstract

This exploratory study assessed parents’ perceptions of the emotional and behavioral impacts of the COVID-19 lockdown on their children. The total sample included 749 children, aged 4 to 13 years old (353 girls, 396 boys); 524 parents took part. The emotional and behavioral changes observed during the societal lockdown, family coexistence, the impact of COVID-19 on family well-being, and the frequency of social contacts before and during this lockdown were investigated. Results show that the most frequently reported difficulties were worry, agitation, anxiety, sadness, loneliness, nervousness, arguing, anger, frustration, boredom, irritability, behavioral problems, and laziness. Family coexistence declined significantly during this lockdown, and parents mentioned that COVID-19 had an impact on family well-being. Various ordinal logistic regressions showed that family coexistence, children’s nervousness due to COVID-19, the impact of COVID-19 on family well-being, age, and social contacts before and during this lockdown seemed to explain the various emotional and behavioral changes observed in children during the societal lockdown. These results are discussed and recommendations are made.

## Introduction

In December 2019, the COVID-19 pandemic, caused by the coronavirus SARS-CoV-2, appeared first in the city of Wuhan (China) before spreading around the whole world a few weeks later. The World Health Organization (WHO) declared a public health emergency of international concern on 30 January 2020, and defined the COVID-19 epidemic as a pandemic on 11 March 2020 (WHO, 2020). Since then, various protective measures have been taken around the world to limit the spread of the virus, which had already caused numerous deaths. These include the implementation of barrier measures such as mask wearing, regular handwashing, and physical distancing. These measures were then escalated as the virus spread farther in the population and lockdowns were declared in many countries. This particular measure resulted in the cancellation of numerous cultural and social events, border closures, countless job losses, forced isolation, very limited movement outside home, etc., resulting in considerable social and economic instability. On 16 March 2020, the governments of Belgium and its neighboring countries announced the closure of schools and all extracurricular activities. The decision to close schools was based on the results of past influenza epidemics, in which school closures reduced social contacts among students and consequently decreased the spread of the virus (Bin Nafisah et al., 2018; Jackson et al., 2016). Nevertheless, this health-related measure has psychological and social consequences. Indeed, school and extracurricular activities are agents of socialization, which (1) foster sociability, improve relationships and feelings of belonging; (2) promote integrity and self-esteem; and (3) provide opportunities to learn (Zarbata et al., 1990). Children learn a variety of social and emotional skills by interacting with their peers (Singh et al., 2020). School routines are important adaptation mechanisms, especially for psychologically fragile children (Lee, 2020).

From a psychological perspective, there is no doubt that COVID-19 has caused its share of problems (Asmundson & Taylor, 2020). Since the start of the pandemic, many researchers around the world have launched studies to understand its causes, consequences and psychological effects. When lockdowns are imposed and lifted in succession, psychological issues appear. In the adults population, several studies have observed symptoms of anxiety, worry, post-traumatic stress and depressive effects further to COVID-19 (e.g., Asmundson & Taylor, 2020; Nelson et al., 2020; Taylor et al., 2020; Torales et al., 2020; Wang et al., 2020; Zhang et al., 2020). Although the psychological impacts of this pandemic on adults are starting to become known (e.g., Torales et al., 2020), there are fewer studies in children. A lockdown to control a virus is quite new in Belgium, as in most countries of the world. Consequently, there is little information in the literature on how it affects children (Orgilés et al., 2020). Understanding children’s reactions, their emotions and the potential effects of this lockdown on their mental health is essential for governments, non-governmental organizations, the community, schools and parents so they can act to reduce the possible effects of this situation and respond correctly to meet children’s needs (Wang et al., 2020).

Since COVID-19 first appeared, several authors have investigated the potential psychological reactions among children and adolescents. In Asian countries, it has been found that younger children (3 to 6 years) were more likely to show symptoms of dependency and fear. Older children and teens (6 to 18 years) were more likely to suffer from inattention and anxiety related to COVID-19. Increased irritability has been revealed in all children, regardless of age group (Viner et al., 2020). Other studies have shown a feeling of isolation, sleep problems, nightmares, loss of appetite, agitation, inattention, and separation anxiety (Bai et al., 2020; Jiao et al., 2020). The closure of schools and colleges had a negative impact on more than 91% of the world’s student population (Lee, 2020). In the European countries, two studies conducted in Italy and Spain showed that most parents observed a psychological impact of the COVID-19 lockdown on their children (Orgilés et al., 2020; Pisano et al., 2020). Changes in the children’s emotional and behavioral conditions were mentioned by 85.7% of the parents (Orgilés et al., 2020). The most frequent symptoms were regressive behaviors (sleep, cleanliness, language); oppositional behaviors (intolerance of rules, excessive demands, agitation, etc.); anxiety and worry; irritability; sleep problems; and depressive affects (apathy, loneliness, boredom, etc.; Orgilés et al., 2020; Pisano et al., 2020). In their study, Pisano et al. (2020) observed that just over 92% of the parents found that their children seemed able to adapt to the pandemic restrictions, even though almost half the children (43.26%) seemed more apathetic about activities they had engaged in regularly before the pandemic, such as playing, or studying. Thus, adaptation is not a sign of wellness. Several authors have pointed out that factors related to the pandemic, such as number of cases per zone or specific lockdown measures (e.g., authorization of some outings), significantly predicted the psychological issues experienced by children and their parents (Bai et al., 2020; Orgilés et al., 2020). Thus, Spanish parents observed more emotional and behavioral symptoms than Italian parents, which may be explained by the fact that young people in Italy had more opportunities to be physically active since they were allowed to go outdoors more often (Orgilés et al., 2020). In a population of Belgian and French children, other authors observed that 35% of the children presented with distress at the time of the first COVID-19 lockdown (Chartier et al., 2021).

Although some studies have highlighted the negative effects of this lockdown, others suggest some positive changes. In fact, in adults, several studies have shown that this COVID-19 crisis has enabled many people to reassess priorities and values (e.g. Sandin et al., 2020). For others, this lockdown allowed to slow down the hectic pace of their daily lives thanks to teleworking, partially unemployment and reduction of activities (Slate.fr, 2020), live a less consuming life (Bouville, 2020), or to spend more time on activities they value (Williams et al., 2021). In children, other authors suggest that this lockdown has allowed, for example, to share more time with their family and get involved in different physical, learning, and creative activities that will help them develop new skills (Gupta & Jawanda, 2020).

Most children’s studies have been done in East Asia, and they may not be generalizable to the rest of the world for cultural, social, and economic reasons (Singh et al., 2020). Thus, the purpose of this exploratory study was to gain a better understanding of both emotional and behavioral impacts of the COVID-19 lockdown on Belgian children to establish the first guidelines on how to reduce the possible effects of this situation, which is nowhere near returning to normal. More specifically, we wanted to investigate: (1) parents’ perception of how the COVID-19 lockdown was affecting their children emotionally and behaviorally; (2) the consequences for family well-being and coexistence; (3) the frequency of social contacts (friends, family, etc.) before the lockdown and their maintenance during it, and how these last two points explained the observed emotional and behavioral changes observed. Ordinal logistic regressions were used to test the findings.

## Methodology

### Participants and procedure

The total sample was composed of 524 Walloon^1^ Belgian parents of children aged between 4 and 13 years old. The participants’ mean age was 40.1 (*SD* = 5.1) and 90.7% of them were women (women: *n* = 475; men: *n* = 49; given this skewness, additional analyzes verified the absence of a significant effect of the parent’s sex variable on the various variables studied.). They were recruited via print media, radio and social media and were invited to respond to an online survey in order to investigate their impressions of their children’s emotional and behavioral condition during the first lockdown. Parents were allowed to complete as many questionnaires as they had children in the age range. The data collected concerns 749 children, of which 47.1% are girls (girls: *n* = 353; boys: *n* = 396); 27% (*n* = 202) were 4 to 5 years old, 31% (*n* = 237) 6 to 8 years old, 25% (*n* = 185) 9 to 11 years old, and 17% (*n* = 125) 12 to 13 years old.

This survey was carried out between 11 and 26 May 2020. All participants took part voluntarily and signed an informed consent form in which they were guaranteed anonymity. This study was approved by the ethics committee of the Faculty of Psychology, Speech Therapy and Educational Sciences of the University of Liège on May 2020 (approval number: 1920-100).

### Measures

To carry out the survey, an ad hoc questionnaire was used based on the study by Orgilés et al. (2020) and an examination of the literature on the psychological impact of Covid-19 lockdown in children. Replicating this Orgilés’ survey in a Belgian population will allow us to compare our data with those obtained in European people.

The questionnaire had five sections: (1) sociodemographic data on the parents and children; (2) parents’ perception of how the lockdown was affecting their children, emotionally and behaviorally, through 32 items measured with a Likert scale ranging from 1 (observed much *less* than before the lockdown) to 5 (observed much *more* than before the lockdown); (3) parents’ perception of family coexistence (*How easy is it to live together as a family?*) before and during the lockdown, measured with a Likert scale ranging from 1 (very easy) to 5 (very difficult), and of the impact of COVID-19 on family well-being, measured on a Likert scale ranging from 1 (no impact at all) to 5 (a really important impact); (4) parents’ perception of their children’s nervousness about the pandemic (when dealing with messages from their parents, the media, etc.), measured with a Likert scale ranging from 1 (never) to 5 (very often); and (5) the frequency of children’s social contacts (friends, family, etc.) before the lockdown and the frequency of social contacts (friends, people outside the family bubble) during the lockdown (digital contact with computer, smartphone, … at the school daycare,^2^ from garden to garden, …), measured with a Likert scale ranging from 1 (never) to 5 (very often).

The first four sections in the questionnaire are similar to those in the study by Orgilés et al. (2020), except second section, in which two items were added (*My child is worried about his/her health* and *My child is lazy (about playing, studying, etc.)*).

### Statistical analyses

All analyses were done with SPSS v.26 and Jamovi v.27. Descriptive statistics were carried out to analyze the participants’ sociodemographic variables and other variables of interest to the study. A Friedman test was used for the repeated-measures analyses of the ordinal variables.

For exploratory purposes, several ordinal logistic regression analyses were used to test the prediction of emotional and behavioral changes observed by parents. This type of analysis was chosen because of the categorical nature of the dependent variables (O’Connell, 2006). In the regression models, the dependent variables introduced were age, sex, family coexistence during the lockdown, impact of COVID-19 on family well-being, and social contacts before and during the lockdown.

## Results

### Parents’ perceptions of changes in their children’s emotional and behavioral condition during the COVID-19 lockdown

During the societal lockdown, most parents observed changes in their children’s emotional and behavioral conditions. The changes most often reported (by at least 30% of parents) were increased worry, anxiety, dependency behaviors, sadness, loneliness, crying, boredom, and laziness. They also mentioned increased nervousness, agitation, reluctance, arguing, anger, irritability, behavioral problems, and difficulties tolerating frustration. From a cognitive point of view, more concentration problems were reported. The detailed percentages of parents reporting changes in their children’s emotional and behavioral condition are presented in ***Table 1***.

**Table 1:** Number (%) of Parents Who Perceived an Emotional and Behavioral Change in Their Children During Covid-19 Lockdown.


	LESS	UNCHANGED	SLIGHTLY MORE	SOMEWHAT MORE	MUCH MORE	TOTAL CHANGE^a^

Worry	23 (3.1%)	340 (45.4%)	247 (33%)	109 (14.6%)	30 (4%)	386 (51.5%)***

Agitation	32 (4.3%)	353 (47.1%)	198 (26.4%)	129 (17.2%)	37 (4.9%)	364 (48.6%)**

Anxiety	31 (4.1%)	417 (55.7%)	179 (23.9%)	101 (13.5%)	21 (2.8%)	301 (40.2%)**

Sadness	35 (4.7%)	387 (51.7%)	187 (25%)	95 (12.7%)	45 (6%)	327 (43.7%)**

Nightmares	26 (3.5%)	606 (80.9%)	73 (9.7%)	28 (3.7%)	16 (2.1%)	117 (15.6%)

Reluctance	15 (2%)	501 (66.9%)	140 (18.7%)	77 (10.3%)	16 (2.1%)	233 (31.1%)*

Loneliness	23 (3.1%)	235 (31.4%)	219 (29.2%)	156 (20.8%)	116 (15.5%)	491 (65.6%)***

Wakefulness	29 (3.9%)	607 (81%)	60 (8%)	36 (4.8%)	17 (2.3%)	113 (15.1%)

Lack of sleep	55 (7.3%)	542 (72.4%)	89 (11.9%)	43 (5.7%)	20 (2.7%)	152 (20.3%)

Indecision	13 (1.7%)	602 (80.4%)	84 (11.2%)	43 (5.7%)	7 (0.9%)	134 (17.9%)

Unease	18 (2.4%)	599 (80%)	89 (11.9%)	36 (4.8%)	7 (0.9%)	132 (17.6%)

Nervousness	27 (3.6%)	371 (49.5%)	206 (27.5%)	111 (14.8%)	34 (4.5%)	351 (46.9%)**

Fear of sleeping	17 (2.3%)	583 (77.8%)	79 (10.5%)	38 (5.1%)	32 (4.3%)	149 (19.9%)

Arguing	36 (4.8%)	384 (51.3%)	196 (26.2%)	95 (12.7%)	38 (5.1%)	329 (43.9%)**

Calm	163 (21.8%)	538 (71.8%)	38 (5.1%)	5 (0.7%)	5 (0.7%)	48 (6.4%)

Crying	25 (3.3%)	474 (63.3%)	157 (21%)	59 (7.9%)	34 (4.5%)	250 (33.4%)*

Anger	26 (3.5%)	394 (52.6%)	201 (26.8%)	94 (12.6%)	34 (4.5%)	329 (43.9%)**

Questions about death	8 (1.1%)	574 (76.6%)	101 (13.5%)	50 (6.7%)	16 (2.1%)	167 (22.3%)

Frustration	15 (2%)	312 (41.7%)	234 (31.2%)	144 (19.2%)	44 (5.9%)	422 (56.3%)***

Boredom	32 (4.3%)	216 (28.8%)	243 (32.4%)	154 (20.6%)	104 (13.9%)	501 (66.9%)***

Irritability	23 (3.1%)	343 (45.8%)	220 (29.4%)	113 (15.1%)	50 (6.7%)	383 (51.1%)***

Difficulty sleeping	22 (2.9%)	537 (71.7%)	102 (13.6%)	51 (6.8%)	37 (4.9%)	190 (25.4%)

Loss of appetite	44 (5.9%)	636 (84.9%)	41 (5.5%)	20 (2.7%)	8 (1.1%)	69 (9.2%)

Alarm	3 (0.4%)	614 (82%)	94 (12.6%)	26 (3.5%)	12 (1.6%)	132 (17.6%)

Concentration	10 (1.3%)	441 (58.9%)	180 (24%)	97 (13%)	21 (2.8%)	298 (39.8%)*

Dependency	83 (11.1%)	401 (53.5%)	143 (19.1%)	84 (11.2%)	38 (5.1%)	265 (35.4%)*

Physical complaints	18 (2.4%)	593 (79.2%)	90 (12%)	33 (4.4%)	15 (2%)	138 (18.4%)

Behavioral problems	30 (4%)	388 (51.8%)	189 (25.2%)	86 (11.5%)	56 (7.5%)	331 (44.2%)**

Eating a lot	43 (5.7%)	517 (69%)	130 (17.4%)	39 (5.2%)	20 (2.7%)	189 (25.2%)

Separation anxiety	13 (1.7%)	559 (74.6%)	124 (16.6%)	38 (5.1%)	15 (2%)	177 (23.6%)

Health worries	3 (0.4%)	595 (79.4%)	120 (16%)	25 (3.3)	6 (0.8%)	151 (20.2%)

Laziness	14 (1.9%)	403 (53.8%)	207 (27.6%)	88 (11.7%)	37 (4.9%)	332 (44.3%)**


*Note: N* = 749, * < 30% of parents mention a change, ** < 40%, *** < 50%. ^a^ Number (%) of parents reporting an emotional or behavioral change (slightly more, somewhat more, or much more).

Regarding the frequency of nervousness felt by the child about the pandemic (due to the restrictions or because the child received messages about COVID-19 from the media or from other people), 32% (*n* = 240) of parents said it had never happened, 29% (*n* = 217) said it had almost never happened, and 30.3% (*n* = 227) reported that it had happened occasionally. The remaining parents, 6.4% (*n* = 48) and 2.3% (*n* = 17), had observed this kind of nervousness in their children quite often or very often, respectively.

### Parents’ perception of family coexistence before and during the lockdown and the impact of COVID-19 on family well-being

Concerning family coexistence (*How easy is it to live together as a family?*), 52.9% of parents reported that it was very easy or easy (respectively, *n* = 124 (16.6%) and *n* = 272 (36.3%)), 34.2% that it was quite easy (*n* = 256), and 12.9% that it was difficult or very difficult (respectively, *n* = 84 (11.2%) and *n* = 13 (1.7%)). When one compares the data from before and during the lockdown, a significant decline in family coexistence is observed (*z* = –12.56; *p* < .001).

As for the impact of the situation caused by COVID-19 on family well-being, 33.3% of parents mentioned no or very little impact (respectively, *n* = 38 (5.1%) and *n* = 211 (28.2%)), 34.4% a moderate impact (*n* = 258), and 32.3% a large or very large impact (respectively, *n* = 200 (26.7%) and *n* = 42 (5.6%)).

### Frequency of social contacts before and during the Covid-19 lockdown

Data concerning the frequency of the children’s social contacts before the lockdown (friends, family, etc.) and the frequency of social contacts (friends, people outside the family bubble) during the lockdown (digital contact with a computer, smartphone, … at the school daycare, from garden to garden, …) are presented in ***Table 2***. The age effect of the children’s social contacts’ frequency during the lockdown was not significant (*p* < .05).

**Table 2:** Frequency (%) of Children’s Social Contacts Before and During Covid-19 Lockdown.


	BEFORE	DURING

Never	0 (0%)	32 (4.3%)

Almost never	9 (1.2%)	195 (26%)

Sometimes	49 (6.5%)	302 (40.3%)

Quite often	255 (34%)	175 (23.4%)

Very often	435 (58.1%)	45 (6%)


### Ordinal logistic regressions

To validate the lack of multicollinearity between independent variables, the values for tolerance and the Variance Inflation Factor (VIF) were calculated. Tolerance values are expected to be greater than .02 and VIF values lower than 10 (Field, 2009). The data are presented in ***Table 3*** and validate the lack of multicollinearity between the predictive variables.

**Table 3:** Results of Multicollinearity Hypothesis Between Independent Variables.


	TOLERANCE	VIF

Age	0.975	1.03

Sex	0.971	1.03

Coexistence	0.816	1.23

Well-being	0.818	1.22

Nervousness	0.937	1.07

Contact 0	0.921	1.09

Contact 1	0.903	1.11


The chi-square test was used to test the validity of the parallel line assumption, which is a second condition for ordinal logistic regression analysis (O’Connell, 2006). The *p* values obtained were greater than .05, which meant that the null hypothesis could be tolerated except for the following items: lack of sleep, unease, nervousness, irritability, physical complaints, and behavioral problems (*p* between .005 and .001). Because the parallel line assumption was violated, no regression analysis was done for these items.

The results of the ordinal logistic regression analysis are presented in ***Table 4***.^3^ Given the exploratory nature of our study, only data concerning the items where a change was perceived by at least 30% of the parents are presented (the other analyses are presented in an appendix). In view of the number of analyses performed, the Bonferroni correction was used to correct the significance threshold, which was set at .001.

**Table 4:** Ordinal Logistic Regression with Score for Child’s Emotional and Behavioral Change as Perceived by the Parents as Dependent Variable.


	WORRY	AGITATION	ANXIETY	SADNESS	RELUCTANCE

Main Effects:					

Age	0.025 (0.025) 1.025 (0.976–1.08)	–0.106* (0.025) 0.900 (0.857–0.945)	0.042 (0.026) 1.043 (0.991–1.098)	–0.021 (0.025) 0.979 (0.932–1.028)	–0.016 (0.028) 0.984 (0.931–1.04)

Sex	–0.134 (0.145) 0.875 (0.658–1.16)	0.438 (0.142) 1.550 (1.175–2.049)	–0.332 (0.149) 0.717 (0.536–0.960)	–0.390 (0.143) 0.677 (0.511–0.895)	0.267 (0.161) 1.306 (0.953–1.79)

Coexistence	0.317* (0.086) 1.373 (1.161–1.62)	0.844* (0.088) 2.326 (1.960–2.771)	0.629* (0.091) 1.875 (1.571–2.245)	0.434* (0.085) 1.543 (1.307–1.824)	0.800* (0.102) 2.226 (1.834–2.72)

Well-being	0.133 (0.083) 1.142 (0.970–1.34)	0.168 (0.081) 1.183 (1.009–1.387)	0.064 (0.086) 1.066 (0.901–1.261)	0.282* (0.081) 1.325 (1.131–1.554)	0.153 (0.096) 1.165 (0.966–1.41)

Nervousness	1.348* (0.088) 3.850 (3.248–4.59)	0.385* (0.072) 1.469 (1.277–1.691)	0.999* (0.083) 2.716 (2.311–3.205)	0.662* (0.076) 1.938 (1.672–2.251)	0.513* (0.081) 1.670 (1.425–1.96)

Contact 0	0.110 (0.113) 1.117 (0.895–1.40)	0.245 (0.110) 1.277 (1.032–1.586)	0.130 (0.113) 1.139 (0.913–1.425)	0.229 (0.111) 1.257 (1.011–1.566)	0.067 (0.121) 1.069 (0.845–1.36)

Contact 1	–0.031 (0.082) 0.969 (0.826–1.14)	–0.028 (0.080) 0.972 (0.832–1.136)	–0.008 (0.083) 0.992 (0.843–1.168)	–0.106 (0.079) 0.900 (0.770–1.051)	–0.092 (0.092) 0.913 (0.762–1.09)

Model fit:					

*X^2^ (df)*	351* (7)	224* (7)	264* (7)	190* (7)	170* (7)

*McFadden’s R^2^*	0.189	0.116	0.151	0.101	0.117

					

	**LONELINESS**	**ARGUING**	**CRYING**	**ANGER**	**FRUSTRATION**

Main Effects:					

Age	0.039 (0.023) 1.039 (0.993–1.088)	–0.008 (0.025) 0.992 (0.946–1.041)	–0.158* (0.028) 0.854 (0.808–0.901)	–0.070 (0.025) 0.932 (0.887–0.979)	0.038 (0.024) 1.039 (0.991–1.09)

Sex	–0.387 (0.134) 0.679 (0.522–0.884)	–0.032 (0.142) 0.968 (0.733–1.279)	–0.117 (0.153) 0.890 (0.659–1.201)	0.094 (0.144) 1.099 (0.829–1.457)	–0.069 (0.139) 0.933 (0.711–1.23)

Coexistence	0.427* (0.080) 1.533 (1.311–1.794)	0.718* (0.088) 2.051 (1.730–2.438)	0.618* (0.095) 1.854 (1.544–2.237)	0.685* (0.089) 1.984 (1.671–2.364)	0.479* (0.083) 1.615 (1.371–1.91)

Well-being	0.219 (0.075) 1.245 (1.074–1.442)	0.220 (0.082) 1.246 (1.062–1.463)	0.086 (0.089) 1.090 (0.915–1.298)	0.160 (0.084) 1.173 (0.996–1.383)	0.248 (0.080) 1.281 (1.095–1.50)

Nervousness	0.507* (0.071) 1.660 (1.445–1.909)	0.334* (0.073) 1.396 (1.210–1.612)	0.395* (0.078) 1.484 (1.274–1.730)	0.509* (0.075) 1.663 (1.437–1.928)	0.589* (0.073) 1.802 (1.564–2.08)

Contact 0	0.515* (0.105) 1.673 (1.363–2.059)	0.192 (0.108) 1.212 (0.982–1.500)	0.074 (0.116) 1.076 (0.858–1.354)	0.154 (0.111) 1.166 (0.939–1.451)	0.494* (0.111) 1.639 (1.320–2.04)

Contact 1	–0.180 (0.075) 0.836 (0.721–0.967)	–0.204 (0.080) 0.816 (0.697–0.953)	0.020 (0.085) 1.020 (0.863–1.204)	–0.023 (0.082) 0.977 (0.833–1.147)	–0.088 (0.078) 0.916 (0.786–1.07)

Model fit:					

*X^2^ (df)*	174* (7)	162* (7)	141* (7)	176* (7)	184* (7)

*McFadden’s R^2^*	0.081	0.087	0.088	0.097	0.095

					

	**BOREDOM**	**CONCENTRATION**	**DEPENDENCY**	**LAZINESS**	

Main Effects:					

Age	0.068 (0.023) 1.071 (1.024–1.120)	0.046 (0.026) 1.047 (0.995–1.10)	–0.110* (0.025) 0.896 (0.853–0.940)	0.163* (0.026) 1.177 (1.119–1.24)	

Sex	0.006 (0.133) 1.006 (0.776–1.306)	0.183 (0.148) 1.201 (0.899–1.61)	0.254 (0.141) 1.290 (0.979–1.700)	0.442 (0.145) 1.556 (1.172–2.07)	

Coexistence	0.610* (0.082) 1.841 (1.568–2.165)	0.464* (0.088) 1.591 (1.340–1.90)	0.450* (0.085) 1.569 (1.328–1.856)	0.429* (0.087) 1.535 (1.297–1.82)	

Well-being	0.156 (0.076) 1.169 (1.007–1.356)	0.057 (0.086) 1.058 (0.894–1.25)	0.056 (0.080) 1.057 (0.904–1.237)	0.199 (0.084) 1.221 (1.035–1.44)	

Nervousness	0.377* (0.069) 1.458 (1.274–1.670)	0.431* (0.075) 1.539 (1.331–1.78)	0.421* (0.072) 1.523 (1.324–1.753)	0.433* (0.075) 1.542 (1.332–1.79)	

Contact 0	0.341* (0.105) 1.406 (1.146–1.727)	0.230 (0.116) 1.259 (1.005–1.58)	0.255 (0.109) 1.290 (1.044–1.599)	0.191 (0.115) 1.211 (0.967–1.52)	

Contact 1	–0.284* (0.075) 0.753 (0.649–0.871)	–0.082 (0.084) 0.921 (0.782–1.09)	–0.025 (0.077) 0.976 (0.838–1.135)	–0.143 (0.082) 0.866 (0.738–1.02)	

Model fit:					

*X^2^ (df)*	163* (7)	95.2* (7)	119* (7)	140* (7)	

*McFadden’s R^2^*	0.075	0.059	0.062	0.080	


*Note: N* = 749; Coexistence = score for difficulty living together; Well-being = score for impact of COVID-19 on family well-being; Nervousness = score for nervousness about COVID-19; Contact 0 = frequency of social contacts before the lockdown; Contact 1 = frequency of maintenance of contact (video or other) during the lockdown. Statistical values: Logistic Coefficient (Standard Error) Odds Ratio (95% CI), * *p* < .001.

All the regression models had a good fit (*p* < .001). The precision of the model’s fit was also tested with McFadden’s pseudo-R^2^, which is considered to be a better index of fit (Allison, 2013). For the various models, the values for McFadden’s pseudo-R^2^ ranged from 0.059 to 0.189. The results of the significance testing of the model’s parameters showed that two variables – coexistence during the lockdown, namely the difficulty of living together with the family, and child’s nervousness about COVID-19, based on information received – significantly predicted all the dependent variables that were analyzed. In addition to those two predictive variables, age significantly predicted agitation, crying, and laziness: younger children presented more agitation and crying, and older children showed more laziness. The existence of increased sadness and loneliness were predicted, in addition to the coexistence problems and nervousness about COVID-19 variables, by frequency of social contacts before the pandemic: children who had more social contacts before the lockdown felt sadder and lonelier during the lockdown. These last three variables, and social contacts during the lockdown significantly predicted boredom, meaning that children who had maintained some social contacts during the lockdown showed less of a change in boredom.

## Discussion

This exploratory study examined the emotional and behavioral changes that parents observed in their children during the lockdown and their impact on family coexistence and well-being. The data suggested that during the first lockdown due to COVID-19, the pandemic substantially impacted children’s emotional and familial well-being. The most important results of this exploratory study will be discussed in the following paragraphs.

Most parents reported a change in their children’s emotional and behavioral condition. The changes most often reported were worry (reported by 51.5% of parents), loneliness (reported by 65.6% of parents), boredom (reported by 66.9% of parents), frustration (reported by 56.3% of parents), and irritability (reported by 51.1% of parents). These changes were observed in more than half of the children. Changes observed in slightly under half of the children were anxiety (reported by 40.2% of parents), nervousness (reported by 46.9% of parents), agitation (reported by 48.6% of parents), arguing (reported by 43.9% of parents), anger (reported by 43.9% of parents), behavioral problems (reported by 44.2% of parents), sadness (reported by 43.7% of parents) and laziness (reported by 44.3% of parents). Finally, reluctance (reported by 31.1% of parents), crying (reported by 33.4% of parents), dependency on adults (reported by 35.4% of parents), and concentration problems (reported by 39.8% of parents) were reported in approximately one-third of children. These results are similar to those reported in the literature (e.g., Jiao et al., 2020; Orgilés et al., 2020; Pisano et al., 2020; Viner et al., 2020).

Family coexistence seems to have deteriorated during this lockdown. In fact, parents reported that it was more difficult to live together as a family. One-third of the parents mentioned that COVID-19 had had a substantial impact on family well-being and another third said that it had had a moderate impact. This increased difficulty getting along might be explained by the obligation to live together under the same roof, resulting in losing an emotional outlet outside the home. This coexistence issue might also result from a series of stressors affecting family members, such as telework, loss of income, homeschooling, or the loss of reinforcement due to the limitation on movements imposed by the lockdown. Although this lockdown inevitably increased the time spent with the family, it placed additional burdens on parents, who were called upon to play several roles unaided, since educational institutions were closed, babysitters and grandparents were unavailable, and contact with peers was not allowed. At the same time, parents were trying to live their own lives and get through their daily workload, which increased the risk of experiencing stress and negative emotions (Griffith, 2020; Spinelli et al., 2020). This situation resulted in many stressors and a high level of pressure, which contributed to developing a climate of stress at home (Cluver et al., 2020; Griffith, 2020). Although we observed no difference between fathers and mothers regarding their perception of family cohesion, a gender bias could be present with a greater perception of cohesion difficulty reported by mothers. The literature reports that more women say they spend more time than their partners on homeschooling during the lockdown than men (Miller, 2020). Literature also reports that gender inequalities in the distribution of household chores may not have diminished (Powell, 2020), or even increased. This could lead to a more negative perception of family cohesion for mothers than for fathers.

Another objective of this study was to investigate the effect of different predictive variables in explaining the emotional and behavioral changes most often reported by parents. The results of the various logistic regressions show that family coexistence during the lockdown and child’s nervousness further to the restrictions and the messages received about COVID-19 are two variables that explained a significant amount of the increase in the emotional and behavioral conditions investigated. The presence of greater difficulty living together as a family would lead to emotional and behavioral changes in the children. Indeed, regarding the predictiveness of family coexistence, previous studies have shown that children’s psychological state is affected by the family environment (Gao et al., 2016). In addition, several studies found that the lockdown had resulted in more family conflicts and affected the parent-child relational experience, which influenced children’s well-being (Spinelli et al., 2020). The risk is that these family conflicts will be expressed as a kind of violence against children, making them vulnerable to emotional damage (Cluver et al., 2020; Jiao et al., 2020; Petito et al., 2020; Solantaus et al., 2004). As for the effect of children’s COVID-19-related nervousness on their psychological condition, the literature mentions that school-aged children are able, to some extent, to assess the crisis, familiar with the use of media, and aware of the negative information the media can convey (Oppenheimer et al., 2016; White, 2017). In this constantly changing situation, the media and social conversations are entirely dominated by the pandemic, and children are exposed to large amounts of information, as well as to the high stress levels of the adults around them (Dalton et al., 2020). Not filtering the information or, conversely, not giving children any information at all can have negative consequences for their psychological wellness. When there is no filter, the risk is that children will receive information that is inappropriate for their age, which creates anxiety. Conversely, children, especially younger ones, tend to rely on their imagination when they do not have enough information to interpret reality, and there is a danger of inappropriate interpretations. Because they are afraid of causing harm, some parents may prefer not to talk about the subject. Nevertheless, children aged two and over are aware of the changes in their environment (Dalton et al., 2019). Indeed, during this pandemic, everyone’s daily life has been greatly modified, the virus has been at the center of everyone’s attention.

The impact of social contacts’ frequency before the lockdown and maintenance of a certain level of social contacts during this was also tested in explaining children’s emotional and behavioral changes. The frequency of social contacts before the lockdown appears to explain the increase in feelings of loneliness, sadness, and frustration. In other words, children who had more social contacts before the lockdown were more affected emotionally. During lockdown, the maintenance of social contacts explained boredom: children who had succeeded in maintaining contacts (by video for example) during the lockdown felt less bored. This health crisis led to the closure of schools and extracurricular activities and restricted movements due to the lockdown, in turn drastically reducing contacts with peers. Interaction with peers is essential to children and teens’ emotional and social well-being (Singh et al., 2020). Thus, the sudden elimination of social contacts has consequences (Buchanan, 2017). In fact, several studies observed an increase in anxiety levels in adults caused by the lack of interpersonal communication due to the lockdown (Kmietowicz, 2020; Xiao, 2020). This effect may also be seen in children. Regarding the frequency of social contacts during the lockdown, we observe that 30% of parents mentioned that their child has never or rarely had social contact (digitally, school daycare, etc) with other children or people outside the family bubble the lockdown. In the digital age, this figure may come as a surprise. However, several explanations are possible such as the more recreational or educational use of technologies by children rather than social use. Indeed, during this lockdown, some schools used digital media to continue learning. As our sample is mainly preschool and primary (and non-secondary) children, the lessons were likely rarely delivered online, resulting in less class interaction. In addition, a survey conducted by the Office for Birth and Childhood (ONE) on the use of technologies in Belgian children can also provide some answers. This report stated that the use of technologies for the purpose of communication and socialization is infrequent among children compared to the period of adolescence; and that such use, when made, requires adult assistance. In a lockdown period where parents have had to face several challenges simultaneously, it is possible that they are not (or little) available to assist their child in digital contacts. This report also mentioned that parents’ attitudes towards screens are quite negative and that they see a little social opportunity (Mathen, 2015). This may have led parents to be less inclined to use this support to maintain social contacts. However, this study was done in children from 0 to 6 years old, which is only part of our sample.

Age was also a significant predictor of the variable agitation, crying and laziness: agitation and crying were more common in younger children, while laziness was observed more frequently in older ones. From a developmental perspective, these observations make sense: crying and agitation are emotional states that occur more often in younger children. Other authors also observed that younger children were more likely to present these symptoms (Singh et al., 2020). As for the increase in laziness, being deprived of varied stimulations over a long period results in a lack of innovative ideas to apply in varied academic and extracurricular activities (Singh et al., 2020). This kind of loss of motivation during the Covid-19 lockdown had also been observed by other authors in older children: they are less motivated to play outside, meet friends or participate in educational activities (Lee, 2020; Liu et al., 2020; Zhai & Du, 2020).

In addition to the variables examined in this study, others might also explain these emotional and behavioral changes observed in children. For example, long-term absence of a structured school framework disrupts routines, which are important adaptation mechanisms for young people (Lee, 2020). Parents’ emotional states could also have an impact on the emotional and behavioral changes observed. In fact, several studies observed that many parents experienced distress during this lockdown (Chartier et al., 2021; Chung et al., 2020); parents’ psychological stress during an epidemic also affects child behavior (e.g., Bai et al., 2020; Chartier et al., 2021; Li et al., 2016; Spinelli et al., 2020; Van Zalk et al., 2018). Moreover, mothers seem to be more impacted than fathers (Chartier et al., 2021; Qiu et al., 2020). So it would be interesting to assess whether this gender effect impacts children differently. As suggested above, the COVID-19 lockdown had consequences on the professional situation of a certain number of parents, such as teleworking, which could have resulted in a parents’ decrease of availability. It would be interesting to investigate the impact of these changes on children’s emotional state and behavior. Furthermore, this study is a cross-sectional study and therefore provides a picture of the situation at a given time. However, the situation regarding COVID-19 is in perpetual flux. Lockdowns are imposed and then lifted again and the situation never remains the same for long. It is therefore essential to continue this kind of investigation and to conduct longitudinal studies to understand more accurately the adaptation (positive and/or negative) of children (but also their adaptation strategies) to the COVID-19 pandemic. Finally, and alongside the negative effects of this crisis, studies should further investigate the possible benefits of it for children. Indeed, several authors suggested positive effects of the lockdown, such as a decrease in the pace of life resulting in a decrease in daily pressures, spending more time doing pleasant things or with family, … However, these possible positive effects of lockdown have been less investigated in children. Additionally, there is a growing evidence base on inequalities associated with COVID-19 (Williams et al., 2021). Several studies highlighted that while some people have had the opportunity to experience the lockdown’s positive effects, others have not (Williams et al., 2021; Wright et al., 2020). This lockdown’s adverse effects appeared to be related to socio-economic factors (household income, education, employment status and housing; Williams et al., 2021). In children and as suggested by Clemens et al. (2020), on the spectrum from healthy–coping to difficulty of coping, many children can expect to suffer, though some will do better. Future studies should investigate these possible inequalities associated with COVID-19 in children.

The various results support several recommendations mentioned in the literature for parents to improve family well-being and decrease the psychological impact of COVID-19 on their children, including the following:

1) Improve family cohesion

Parents could assess the level of family coexistence at regular intervals so they can quickly identify any difficulties affecting family cohabitation and act accordingly, for example by taking some time for themselves. When parents feel that they are unable to be available for their children, they may decide to hand over briefly to another available adult who is able to pay sensitive, coherent attention to the children (Bartlett et al., 2020). Concerning policy recommendations, particular attention should be paid to the prevention, and promotion of family cohesion. Providing online questionnaire to detect psychological distress in the family, giving access to several free resources, and providing psychoeducation to parents to help them identify early warning signs of familial cohesion deterioration, as well as early regulatory strategies, will also help mitigate negative outcomes of the pandemic.

2) Communicate appropriately

When dealing with children’s concerns and questions, it is important for adults to be open to communication. Communication must be appropriate for the children’s age and bear in mind their understanding of the disease and its causes (Dalton et al., 2020). To do this, parents can use books, explanatory brochures,^4^ as well as the websites which present information on COVID-19 adapted for children (Bartlett et al., 2020; Singh et al., 2020): for example Covidforkids^5^ or Watwat,^6^ and these provided by the WHO^7^ and UNICEF.^8^ Between the ages of 4 and 7, children overestimate the impact of their own behavior on the appearance of a disease (Edwards & Davis, 1997). Parents should therefore make sure that children do not blame themselves inappropriately or think that the disease is a punishment for bad behavior. Honest communication should provide a coherent explanation of what is being observed and help children normalize their emotional reactions to COVID-19 (Dalton et al., 2020). Children’s exposure to social media and to adults’ conversations about the pandemic should be limited because these channels are less adapted to their age (Bartlett et al., 2020; Singh et al., 2020).

3) Maintain social contacts

Social ties improve children’s resiliency in the face of complex situations. Creative approaches to remaining connected are therefore essential (e.g., letters, video conversations, etc.; Bartlett et al., 2020). They are particularly important when the frequency of the child’s social contacts before COVID-19 was high. Concerning policy recommendations, a reflection on the development of digital literacy should be done in order to promote the maintenance of social contacts even in a period of lockdown. Social distancing should not be synonymous with social isolation. At the level of schools, teachers should reflect on their teaching by integrating a maximum of virtual interaction between the teacher and the pupils but also the pupils between them. Moreover, it’s important to provide more information about the relative risks and benefits of virtual social contact to parents who overestimate the dangers of allowing their children too much screen time (Loades et al., 2020).

4) Create a secure emotional environment

Many authors mention the importance of maintaining a routine to give children a feeling of security and predictability, for example by setting regular bedtimes and mealtimes and daily schedules for learning and play (Bartlett et al., 2020; Singh et al., 2020). When children get bored, their level of anxiety and disruptive behaviors can increase. In that case, it is essential for parents to maintain a certain level of activity for their children by suggesting various recreational activities (Bartlett et al., 2020) and providing a stimulating environment to counteract laziness and loss of motivation (Singh et al., 2020). Children’s well-being also depends on their parents’ well-being. To maintain the internal resources they need to look after their children, parents must take care of themselves, for example by maintaining social contacts or taking the time for restorative activities (Bartlett et al., 2020).

5) Seek help from professionals

In the current situation, emotional and behavioral changes are inevitable. Nevertheless, if these changes are substantial and persistent and constantly disrupt good family functioning, and if they are not resolved with support, professional help may be necessary (Bartlett et al., 2020). Many mental health care providers offer teleconsultation services that can include psychoeducation and first-line support^9^ (Galea et al., 2020; Singh et al., 2020).

Although this study has presented some interesting results, it is affected by certain limitations. First of all, only the parents’ perceptions were investigated and not the children’s. The parents’ own psychological state may have influenced how they answered. Secondly, the sample was composed mainly of mothers, introducing a possible gender bias. Although our study does not highlight an effect of parent gender in the reported responses, several studies have shown that the latter were more particularly impacted emotionally by this lockdown (Chartier et al., 2021; Qiu et al., 2020). Thirdly, sampling bias is possible in light of the recruitment of participants via the media and the administration of online surveys. The profile of the “internet-savvy” population is not the same as that of the general population, even though this aspect is changing. Fourthly, this is a cross-sectional study and therefore provides a picture of the situation depending on lockdown measures at a given time. This study cannot, at this stage, provide that these changes were maintained over time. Finally, the number of statistical analyses obliged us to apply a stringent correction of *p* values; consequently, some effects may not have been highlighted.

In conclusion, the COVID-19 pandemic and the consequent lockdown had an impact on Belgian children’s emotional and behavioral status and on family coexistence. Several variables appear to predict these changes, namely family coexistence, children’s nervousness about COVID-19, and social contacts. The results of this study provide new information in this research area and also suggest avenues for preventive action by parents to mitigate the psychological effects of this pandemic on their children. It is crucial to continue studying this topic, in view of the ever-changing situation.

## Additional File

The additional file for this article can be found as follows:

10.5334/pb.1059.s1 Supplemental Materials 1. *Appendix*. Ordinal logistic regression with the child’s emotional and behavioral change score perceived by the parents as a dependent variable.

## Footnotes

People living in Wallonia, one of the three regions of Belgium and which is made up of the provinces of Walloon Brabant, Hainaut, Liège, Luxembourg and Namur.

In Belgium, a school daycare was organized for parents who had a profession in the medical sector or who had to go to their workplace.

When the parent’s gender variable is introduced as a covariate, the result of pattern remains absolutely the same.

*https://www.chuliege.be/jcms/c2_20509209/fr/direction-medicale/le-covid-19-explique-aux-enfants*; *https://assets.watwat.be/attachment/MAGDANOG-brochure-Frans.pdf*.

*covidforkids.info*.

*https://www.watwat.be/corona*.

*https://www.who.int/fr/news/item/09-04-2020-children-s-story-book-released-to-help-children-and-young-people-cope-with-covid-19*.

*https://www.unicef.org/fr/covid-19-et-masques-conseils-destinés-aux-familles*.

*http://www.psy107.be/index.php/fr/conventie-klin-psy-orth*.
