# Supplementary material for: Parents’ Perceived Impact of the Societal Lockdown of COVID-19 on Family Well-Being and on the Emotional and Behavioral State of Walloon Belgian Children Aged 4 to 13 Years: An Exploratory Study
Source: Psychol Belg. 2021 Jun 29;61(1):186–99. doi: 10.5334/pb.1059 (PMC8252974; doi:10.5334/pb.1059)
Supplement: Supplemental Materials 1. — Appendix. Ordinal logistic regression with the child’s emotional and behavioral change score perceived by the parents as a dependent variable. [file pb-61-1-1059-s1.pdf]

Supplementary file 1: Appendix. Ordinal logistic regression with the child's emotional and behavioral change score perceived by the parents as a dependent variable

	Worry	Agitation	Anxiety	Sadness	Nightmares
Main Effects :					
Age	0.025 (0.025) 1.025 (0.976-1.08)	**-0.106* (0.025) 0.900 (0.857-0.945)**	0.042 (0.026) 1.043 (0.991-1.098)	-0.021 (0.025) 0.979 (0.932-1.028)	-0.099 (0.034) 0.906 (0.847-0.968)
Sex	-0.134 (0.145) 0.875 (0.658-1.16)	0.438 (0.142) 1.550 (1.175-2.049)	-0.332 (0.149) 0.717 (0.536-0.960)	-0.390 (0.143) 0.677 (0.511-0.895)	-0.500 (0.192) 0.607 (0.415-0.882)
Coexistence	**0.317* (0.086) 1.373 (1.161-1.62)**	**0.844* (0.088) 2.326 (1.960-2.771)**	**0.629* (0.091) 1.875 (1.571-2.245)**	**0.434* (0.085) 1.543 (1.307-1.824)**	0.301 (0.111) 1.352 (1.090-1.685)
Well-being	0.133 (0.083) 1.142 (0.970-1.34)	0.168 (0.081) 1.183 (1.009-1.387)	0.064 (0.086) 1.066 (0.901-1.261)	**0.282* (0.081) 1.325 (1.131-1.554)**	0.167 (0.109) 1.182 (0.954-1.464)
Nervousness Covid	**1.348* (0.088) 3.850 (3.248-4.59)**	**0.385* (0.072) 1.469 (1.277-1.691)**	**0.999* (0.083) 2.716 (2.311-3.205)**	**0.662* (0.076) 1.938 (1.672-2.251)**	**0.500* (0.097) 1.648 (1.365-1.995)**
Contact 0	0.110 (0.113) 1.117 (0.895-1.40)	0.245 (0.110) 1.277 (1.032-1.586)	0.130 (0.113) 1.139 (0.913-1.425)	0.229 (0.111) 1.257 (1.011-1.566)	-0.043 (0.146) 0.958 (0.722-1.282)
Contact 1	-0.031 (0.082) 0.969 (0.826-1.14)	-0.028 (0.080) 0.972 (0.832-1.136)	-0.008 (0.083) 0.992 (0.843-1.168)	-0.106 (0.079) 0.900 (0.770-1.051)	-0.275 (0.109) 0.760 (0.613-0.940)
Model fit :					
X^2	**351* (7)**	**224* (7)**	**264* (7)**	**190* (7)**	**82.4 (7)**
McFadden R^2	0.189	0.116	0.151	0.101	0.077

	Reluctance	Solitude	Waking up	Indecision	Fear of sleeping
Main Effects :					
Age	-0.016 (0.028) 0.984 (0.931-1.04)	0.039 (0.023) 1.039 (0.993-1.088)	-0.017 (0.033) 0.983 (0.921-1.048)	0.016 (0.033) 1.016 (0.952-1.08)	**-0.126* (0.033) 0.882 (0.827-0.940)**
Sex	0.267 (0.161) 1.306 (0.953-1.79)	-0.387 (0.134) 0.679 (0.522-0.884)	-0.422 (0.190) 0.656 (0.450-0.950)	0.243 (0.190) 1.275 (0.880-1.86)	-0.313 (0.181) 0.731 (0.512-1.043)
Coexistence	**0.800* (0.102) 2.226 (1.834-2.72)**	**0.427* (0.080) 1.533 (1.311-1.794)**	0.321 (0.112) 1.379 (1.110-1.721)	**0.537* (0.116) 1.711 (1.367-2.16)**	0.203 (0.104) 1.225 (1.000-1.507)
Well-being	0.153 (0.096) 1.165 (0.966-1.41)	0.219 (0.075) 1.245 (1.074-1.442)	0.053 (0.108) 1.054 (0.853-1.304)	0.079 (0.113) 1.082 (0.867-1.35)	0.080 (0.104) 1.083 (0.883-1.326)
Nervousness Covid	**0.513* (0.081) 1.670 (1.425-1.96)**	**0.507* (0.071) 1.660 (1.445-1.909)**	**0.404* (0.095) 1.498 (1.244-1.807)**	**0.554* (0.096) 1.741 (1.443-2.11)**	**0.622* (0.092) 1.862 (1.558-2.234)**
Contact 0	0.067 (0.121) 1.069 (0.845-1.36)	**0.515* (0.105) 1.673 (1.363-2.059)**	0.003 (0.147) 1.003 (0.754-1.343)	0.020 (0.143) 1.020 (0.775-1.36)	0.055 (0.140) 1.056 (0.805-1.397)
Contact 1	-0.092 (0.092) 0.913 (0.762-1.09)	-0.180 (0.075) 0.836 (0.721-0.967)	-0.171 (0.107) 0.843 (0.683-1.039)	-0.121 (0.107) 0.885 (0.717-1.09)	-0.096 (0.102) 0.909 (0.744-1.109)
Model fit :					
X^2	**170* (7)**	**174* (7)**	**48.4* (7)**	**83* (7)**	**87.1* (7)**
McFadden R^2	0.117	0.081	0.045	0.079	0.072

	Argument	Calm	Crying	Anger	Question about death
Main Effects :					
Age	-0.008 (0.025) 0.992 (0.946-1.041)	0.026 (0.029) 1.027 (0.970-1.087)	**-0.158* (0.028) 0.854 (0.808-0.901)**	-0.070 (0.025) 0.932 (0.887-0.979)	**-0.292* (0.038) 0.747 (0.693-0.802)**
Sex	-0.032 (0.142) 0.968 (0.733-1.279)	-0.185 (0.167) 0.831 (0.598-1.152)	-0.117 (0.153) 0.890 (0.659-1.201)	0.094 (0.144) 1.099 (0.829-1.457)	-0.475 (0.186) 0.622 (0.431-0.892)
Coexistence	**0.718* (0.088) 2.051 (1.730-2.438)**	**-0.682* (0.102) 0.506 (0.413-0.616)**	**0.618* (0.095) 1.854 (1.544-2.237)**	**0.685* (0.089) 1.984 (1.671-2.364)**	0.088 (0.105) 1.091 (0.889-1.344)
Well-being	0.220 (0.082) 1.246 (1.062-1.463)	-0.092 (0.094) 0.912 (0.758-1.095)	0.086 (0.089) 1.090 (0.915-1.298)	0.160 (0.084) 1.173 (0.996-1.383)	-0.018 (0.103) 0.982 (0.800-1.201)
Nervousness Covid	**0.334* (0.073) 1.396 (1.210-1.612)**	-0.160 (0.083) 0.853 (0.724-1.004)	**0.395* (0.078) 1.484 (1.274-1.730)**	**0.509* (0.075) 1.663 (1.437-1.928)**	**0.862* (0.098) 2.368 (1.960-2.881)**
Contact 0	0.192 (0.108) 1.212 (0.982-1.500)	-0.407 (0.130) 0.665 (0.514-0.858)	0.074 (0.116) 1.076 (0.858-1.354)	0.154 (0.111) 1.166 (0.939-1.451)	-0.109 (0.141) 0.897 (0.681-1.186)
Contact 1	-0.204 (0.080) 0.816 (0.697-0.953)	0.173 (0.092) 1.189 (0.993-1.425)	0.020 (0.085) 1.020 (0.863-1.204)	-0.023 (0.082) 0.977 (0.833-1.147)	0.023 (0.106) 1.023 (0.832-1.258)
Model fit :					

X^2	**162* (7)**	**93.9* (7)**	**141* (7)**	**176* (7)**	**160* (7)**
McFadden R^2	0.087	0.080	0.088	0.097	0.136

	Frustration	Boredom	Difficulty sleeping	No appetite	Alarming
Main Effects :					
Age	0.038 (0.024) 1.039 (0.991-1.09)	0.068 (0.023) 1.071 (1.024-1.120)	0.035 (0.029) 1.036 (0.979-1.096)	-0.022 (0.035) 0.978 (0.913-1.048)	-0.051 (0.037) 0.950 (0.883-1.02)
Sex	-0.069 (0.139) 0.933 (0.711-1.23)	0.006 (0.133) 1.006 (0.776-1.306)	-0.338 (0.165) 0.714 (0.516-0.984)	0.083 (0.203) 1.087 (0.730-1.621)	0.167 (0.208) 1.182 (0.787-1.78)
Coexistence	**0.479* (0.083) 1.615 (1.371-1.91)**	**0.610* (0.082) 1.841 (1.568-2.165)**	0.313 (0.096) 1.368 (1.134-1.655)	0.134 (0.119) 1.144 (0.905-1.446)	**0.523* (0.129) 1.687 (1.315-2.18)**
Well-being	0.248 (0.080) 1.281 (1.095-1.50)	0.156 (0.076) 1.169 (1.007-1.356)	0.150 (0.094) 1.162 (0.966-1.399)	-0.022 (0.114) 0.979 (0.783-1.224)	-0.205 (0.129) 0.815 (0.631-1.05)
Nervousness Covid	**0.589* (0.073) 1.802 (1.564-2.08)**	**0.377* (0.069) 1.458 (1.274-1.670)**	**0.452* (0.084) 1.571 (1.333-1.854)**	0.267 (0.101) 1.306 (1.072-1.590)	**1.319* (0.121) 3.738 (2.963-4.78)**
Contact 0	**0.494* (0.111) 1.639 (1.320-2.04)**	**0.341* (0.105) 1.406 (1.146-1.727)**	0.304 (0.134) 1.355 (1.046-1.771)	-0.303 (0.151) 0.739 (0.551-0.996)	-0.111 (0.158) 0.895 (0.659-1.23)
Contact 1	-0.088 (0.078) 0.916 (0.786-1.07)	**-0.284* (0.075) 0.753 (0.649-0.871)**	-0.094 (0.092) 0.910 (0.759-1.090)	0.094 (0.113) 1.098 (0.880-1.369)	0.171 (0.120) 1.187 (0.937-1.50)
Model fit :					
X^2	**184* (7)**	**163* (7)**	**78.6* (7)**	13.8 (7)	**179* (7)**
McFadden R^2	0.095	0.075	0.056	0,015	0.191

	Concentration	Dependence	Eating a lot	Worried separation	Worried health
Main Effects :					
Age	0.046 (0.026) 1.047 (0.995-1.10)	-0.110* (0.025) 0.896 (0.853-0.940)	0.047 (0.027) 1.048 (0.995-1.10)	**-0.187* (0.033) 0.829 (0.776-0.884)**	0.053 (0.034) 1.054 (0.985-1.13)
Sex	0.183 (0.148) 1.201 (0.899-1.61)	0.254 (0.141) 1.290 (0.979-1.700)	-0.131 (0.156) 0.877 (0.646-1.19)	-0.098 (0.175) 0.906 (0.642-1.278)	-0.107 (0.196) 0.898 (0.612-1.32)
Coexistence	**0.464* (0.088) 1.591 (1.340-1.90)**	**0.450* (0.085) 1.569 (1.328-1.856)**	0.055 (0.091) 1.056 (0.884-1.26)	-0.096 (0.100) 0.908 (0.747-1.105)	0.015 (0.114) 1.015 (0.813-1.27)
Well-being	0.057 (0.086) 1.058 (0.894-1.25)	0.056 (0.080) 1.057 (0.904-1.237)	0.053 (0.087) 1.055 (0.889-1.25)	0.148 (0.096) 1.160 (0.960-1.401)	-0.068 (0.114) 0.934 (0.746-1.17)
Nervousness Covid	**0.431* (0.075) 1.539 (1.331-1.78)**	**0.421* (0.072) 1.523 (1.324-1.753)**	0.074 (0.079) 1.077 (0.922-1.26)	**0.862* (0.092) 2.367 (1.980-2.844)**	**1.194* (0.115) 3.299 (2.648-4.16)**
Contact 0	0.230 (0.116) 1.259 (1.005-1.58)	0.255 (0.109) 1.290 (1.044-1.599)	0.093 (0.121) 1.097 (0.867-1.39)	-0.101 (0.132) 0.904 (0.699-1.174)	0.038 (0.152) 1.039 (0.773-1.41)
Contact 1	-0.082 (0.084) 0.921 (0.782-1.09)	-0.025 (0.077) 0.976 (0.838-1.135)	-0.104 (0.088) 0.902 (0.758-1.07)	-0.025 (0.098) 0.975 (0.804-1.182)	-0.069 (0.114) 0.934 (0.746-1.17)
Model fit :					
X^2	**95.2* (7)**	**119* (7)**	8.27 (7)	**132* (7)**	**149* (7)**
McFadden R^2	0.059	0.062	0.006	0.109	0.153

	Laziness
Main Effects :	
Age	**0.163* (0.026) 1.177 (1.119-1.24)**
Sex	0.442 (0.145) 1.556 (1.172-2.07)
Coexistence	**0.429* (0.087) 1.535 (1.297-1.82)**
Well-being	0.199 (0.084) 1.221 (1.035-1.44)
Nervousness Covid	**0.433* (0.075) 1.542 (1.332-1.79)**
Contact 0	0.191 (0.115) 1.211 (0.967-1.52)
Contact 1	-0.143 (0.082) 0.866 (0.738-1.02)
Model fit :	
X^2	**140* (7)**

McFadden R^2	0.080

Note. *N* = 749; Coexistence = score for difficulty living together; Well-being = score for impact of COVID-19 on family well-being; Nervousness = score for nervousness about COVID-19; Contact 0 = frequency of social contacts before the lockdown; Contact 1 = frequency of maintenance of contact (video or other) during the lockdown.

Statistical values: Logistic Coefficient (Standard Error) Odds Ratio (95% CI), $*p < .001$
